# Metabolic modeling to identify engineering targets for *Komagataella phaffii*: The effect of biomass composition on gene target identification

**DOI:** 10.1002/bit.26380

**Published:** 2017-08-15

**Authors:** Ayca Cankorur‐Cetinkaya, Duygu Dikicioglu, Stephen G. Oliver

**Affiliations:** ^1^ Cambridge Systems Biology Centre & Department of Biochemistry University of Cambridge Cambridge UK

**Keywords:** biomass composition, genome‐scale metabolic model, *Komagataella phaffii*, metabolic target identification, *Pichia pastoris*, recombinant protein production

## Abstract

Genome‐scale metabolic models are valuable tools for the design of novel strains of industrial microorganisms, such as *Komagataella phaffii* (syn. *Pichia pastoris*). However, as is the case for many industrial microbes, there is no executable metabolic model for *K. phaffiii* that confirms to current standards by providing the metabolite and reactions IDs, to facilitate model extension and reuse, and gene‐reaction associations to enable identification of targets for genetic manipulation. In order to remedy this deficiency, we decided to reconstruct the genome‐scale metabolic model of *K. phaffii* by reconciling the extant models and performing extensive manual curation in order to construct an executable model (Kp.1.0) that conforms to current standards. We then used this model to study the effect of biomass composition on the predictive success of the model. Twelve different biomass compositions obtained from published empirical data obtained under a range of growth conditions were employed in this investigation. We found that the success of Kp1.0 in predicting both gene essentiality and growth characteristics was relatively unaffected by biomass composition. However, we found that biomass composition had a profound effect on the distribution of the fluxes involved in lipid, DNA, and steroid biosynthetic processes, cellular alcohol metabolic process, and oxidation‐reduction process. Furthermore, we investigated the effect of biomass composition on the identification of suitable target genes for strain development. The analyses revealed that around 40% of the predictions of the effect of gene overexpression or deletion changed depending on the representation of biomass composition in the model. Considering the robustness of the in silico flux distributions to the changing biomass representations enables better interpretation of experimental results, reduces the risk of wrong target identification, and so both speeds and improves the process of directed strain development.

## 1 INTRODUCTION

The use of genetic manipulation to modify specific cellular biochemical reactions or to introduce new ones is a common approach in the directed improvement of microbial strains used in biotechnological processes. In order to achieve this, a comprehensive understanding of the metabolic network of the “chassis” organism is required in order to identify the most promising targets to be modified. The availability of complete genome sequences and advances in systems biology allow the analysis of large datasets in an integrated manner to increase our understanding of metabolism and enable model‐driven strain design strategies. Genome‐scale metabolic models (GEMs) can be used to predict the metabolic capabilities of a “chassis” organism, to investigate the changes in the flux distributions under different conditions, to identify metabolic bottlenecks, and to evaluate the outcomes of the possible genetic modifications (Durot, Bourguignon, & Schachter, 2009; Kim et al., 2012; Nielsen & Keasling, 2016; Ostergaard, Olsson, & Nielsen, 2000; Stephanopoulos, Aristidou, & Nielsen, 1998).

The predictive accuracy of GEMs varies greatly; for instance, their use to predict genetic interactions provides an accurate picture of the topology of the interaction network, while failing to predict most of the individual interactions revealed in high‐throughput experiments (Szappanos et al., 2011). Therefore, if the predictive accuracy of these models is to be improved, they must be continuously updated in the light of current knowledge. Manual curation continues to play an important role in refining and improving GEMS and, if the whole research community is to participate in this process, standard formats (such as, Systems Biology Mark‐up Language, SBML; Hucka et al., 2003) must be employed to present these models. The use of SBML in combination with the annotation of metabolites, enzymes, and genes with database IDs facilitates the reuse of models, their extension, and comparisons between different GEMs for the same species (Ravikrishnan & Raman, 2015). The construction of a consensus metabolic network model for *Saccharomyces cerevisiae* represents a good example of how the community can continue to update the model once a reusable consensus network has been constructed (Herrgård et al., 2008).

*Komagataella phaffii* (*Pichia pastoris*) is a well‐established host organism used for recombinant protein production (Demain & Vaishnav, 2009). Many researchers have worked on improving the productivity of this host organism by genetic manipulation, usually by designing new expression vectors or optimizing the copy number of the transgene (Idiris, Tohda, Kumagai, & Takegawa, 2010; Prielhofer et al., 2013; Puxbaum, Mattanovich, & Gasser, 2015; Shen et al., 2016). A GEM *for K. phaffii* would represent a valuable tool for the in silico testing of possible genetic modifications designed to improve the yield or efficiency of biotechnological processes that employ this industrially important yeast. There are three independently constructed GEMs of this organism (Caspeta, Shoaie, Agren, Nookaew, & Nielsen, 2012; Chung et al., 2010; Sohn et al., 2010). Although these models have potential for the in silico identification of genetic modification targets (Nocon et al., 2014) or the development of new cultivation strategies (Irani, Maghsoudi, Shojaosadati, & Motamedian, 2015), their lack of standard annotation limits our ability to compare and analyze these models and also to improve these models in the light of current information. Moreover, these models do not make use of valuable information about the biomass composition of the *K. phaffii*, which has been garnered under a range of experimental conditions (Carnicer et al., 2009; Jordà, de Jesus, Peltier, Ferrer, & Albiol, 2014).

In this study, we first reconstructed the metabolic network of *K. phaffii* through extensive manual curation in order to obtain a reusable model (Kp.1.0) comprising the standard metabolite and reaction IDs and gene‐reaction associations that permit the in silico simulations and analyses. We next investigated the effect of biomass representation on the predictive capability of Kp.1.0 by using published biomass composition datasets for this organism. We then focused on how the changing biomass representations affected the models predictions on targets to be engineered to increase this yeast's productivity. In all, our study has investigated the potential of a new GEM in identifying targets for improving the productivity of *K. phaffii* cells.

## 2 MATERIALS AND METHODS

### 2.1 Model comparison

We set out to unify the three independently constructed genome‐scale metabolic models of *K. phaffii*; PpaMBEL1254 (Sohn et al., 2010), iPP668 (Chung et al., 2010), and ILC915 (Caspeta et al., 2012), initially by employing the same nomenclatures for cell compartments and metabolites. For metabolites, all available KEGG (Kanehisa, Sato, Kawashima, Furumichi, & Tanabe, 2016; Kanehisa & Goto, 2000) and ChEBI (Hastings et al., 2013) ID associations of all the metabolites in these models were identified. For instances where the metabolite name used in the model was not encountered in any database, the reactions involved were curated manually and appropriate IDs assigned. Manual comparisons of the reactions were then conducted, taking into consideration the differences due to directionality, representation of currency metabolites, or compartmentalization.

### 2.2 Network reconstruction

A core network was constructed using all reactions that were found in at least two of the three models. *K. phaffii* metabolic pathways in the KEGG and the MetaCyc (Caspi et al., 2014) databases were used to resolve any discrepancies in reaction directionality, representation of currency metabolites, and compartmentalization. Enzyme‐gene associations were also taken from the KEGG. Reactions were annotated with KEGG reaction IDs and genes/proteins were annotated with UniProt IDs (UniProt Consortium, 2008). Cellular compartment assignments were made using “Gene Ontology” terms whenever available in (Sterck, Billiau, Abeel, Rouzé, & Van de Peer, 2012) or were based on orthology with *S. cerevisiae* (Nash et al., 2007). Dead‐end metabolites (i.e., those that were produced but not used as substrates in any reaction, or those that were used as substrate but not produced in any reaction) were identified and any resulting gaps in the network filled by manual curation.

### 2.3 Representation of biomass formation

The Kp.1.0 model contains 12 different biomass equations that account for recombinant protein production, different aeration levels, different ratios of glycerol:methanol co‐feeding, and different dilution rates in continuous cultures. The data regarding the wild‐type and recombinant strains (producing heterologous proteins, namely the antibody fragment, Fab) grown under different oxygenation levels (normoxic, oxygen‐limited, and hypoxic) were adopted from (Carnicer et al., 2009). The biomass composition of a *K. phaffii* strain producing *Rhizopus oryzae* lipase (ROL), and grown aerobically in chemostat cultures fed with 80:20, 60:40, and 40:60 (w:w) glycerol/methanol mixtures at two dilutions rates (0.05 and 0.16 hr^−1^) were obtained from Jordà et al. (2014). The biomass compositions were represented as the aggregate of the carbohydrates, proteins, lipids, RNA, DNA, as well as the ATP consumption associated with the growth. Furthermore, Carnicer et al. (2009) reported the amino‐acid composition of the protein macromolecules and the glycogen and trehalose content of carbohydrate macromolecule under corresponding conditions and Jordà et al. reported the amino‐acid composition of the aggregated protein macromolecules at each dilution rate examined. These datasets were also incorporated into the model to account for the content change of proteins and polysaccharides, whenever the data were available.

### 2.4 Model construction, simulations, and the analyses of the results

Kp.1.0 (File S1) was prepared as a COBRA‐compliant SBML version 2, level 4 (Finney & Hucka, 2003; Hucka et al., 2003) in compliance with the MIRIAM guidelines (Laibe & Le Novère, 2007). Analyses were conducted using the COBRA tool‐box (v.2.0.5) under MATLAB R2013b (8.2.0.701 Mathworks; Natick, MA) (Schellenberger et al., 2011) with SBML Toolbox v4.1.0 and libSBML library v5.5.0 using the Gurobi5 solver. Maximization of biomass production was used as the objective function in all simulations, unless otherwise stated. The experimental data used to constrain the model in order to predict the growth and carbon dioxide exchange rates (CER) under each defined condition are given in Table 1. Changes in flux distributions due to genetic mutations (deletions) were investigated employing the FBA algorithm and by constraining the glucose uptake rate (alone) to unity. The predictive ability of the model was evaluated using phenotypic data for the *S. cerevisiae* orthologs of *K. phaffii* genes (Table 2).

**Table 1:** Datasets used to constrain the model^a^

	*q*_Glu_	*q*_Met_	*q*_Gly_	$q_{O_{2}}$	*q*_EtOH_	*q*_Ara_	*q*_Fab_	
Carnicer et al. (2009)								
Wild type								
Normoxic	0.99	n/a	n/a	2.35	n/a	n/a	n/a	
Oxygen‐limited	1.28	n/a	n/a	2.01	0.31	0.13	n/a	
Hypoxic	1.72	n/a	n/a	2.01	0.84	0.33	n/a	
Fab producing								
Normoxic	1.01	n/a	n/a	2.44	n/a	n/a	0.0004	
Oxygen‐limited	1.37	n/a	n/a	1.99	0.41	0.19	0.0007	
Hypoxic	1.56	n/a	n/a	1.81	0.83	0.24	0.0007	
Rußmayer et al. (2015)								
Wild type								
Grown on Glu	1.02	n/a	n/a	2.39	n/a	n/a	n/a	
Grown on Met + Gly	n/a	0.81	1.64	3.09	n/a	n/a	n/a	

^a^ Glu, Met, Gly, O2, EtOH, Ara, and Fab denote glucose, methanol, glycerol, oxygen, ethanol, arabitol and antibody Fab fragment, respectively. *q* denotes specific utilization rates for glucose, methanol, glycerol, oxygen, and specific production rates for ethanol, arabitol and antibody Fab fragment and given in mmol/gCDW/hr.

**Table 2:** Evaluation of the predictive ability of the gene essentiality

Kp.1.0 prediction for deletion of *K. phaffii* gene	Deletion mutant phenotype of *S. cerevisiae* ortholog	
Viable	Viable	True positive (TP)
Viable	Inviable	False positive (FP)
Inviable	Inviable	True negative (TN)
Inviable	Viable	False negative (FN)

The significance of the effect of biomass composition on the flux distributions was assessed by using both Mann–Whitney *U* test (*p*‐value < 0.01) and fold change (FC > 2) analyses. For each case, 100 random values for each flux in the distribution were generated such that each value remained within bounds of the allowable range for that flux, which was determined through flux variability analysis. The Mann–Whitney *U* test was applied to those values for each individual flux separately, in order to identify those changes, which have appreciable biological impact, the average of the 100 values for each individual flux were employed in all possible pairwise comparisons in fold change analyses. A similar analysis was also conducted for sample sizes of 50, 1,000, and 10,000 to test the effect of the sample size on the significance analysis. Flux values lower than 10^−5^ were taken as zero. The significance of between‐biomass comparisons under different conditions was calculated using Mann–Whitney *U* test for each macromolecule.

Princeton GO Tools were used to conduct the GO‐term enrichment analysis employing GO Term Finder (Boyle et al., 2004). The .obo gene ontology mapping file and *K. phaffii* annotation file (34378.P_pastoris_GS115.obo) were downloaded from EMBL‐EBI (http://www.ebi.ac.uk) on March 29, 2016. Orthologous genes were obtained from InParanoid 8 database, considering only data that have 100% bootstrap confidence value (Sonnhammer & Östlund, 2015).

Flux Scanning based Enforced Objective Function (FSEOF) (Choi, Lee, Kim, & Woo, 2010) was used to identify the overexpression targets that would increase human copper/zinc superoxide dismutase (hSOD) production. The reactions that were catalyzed by isoenzymes and promiscuous enzymes were not considered as candidates. RobOKoD (Stanford, Millard, & Swainston, 2015) was employed to identify potential knockout targets for overproduction of hSOD. Similarly, only the reactions that have 1:1 gene‐reactions mappings were considered.

## 3 RESULTS

### 3.1 Manual curation is necessary to generate a consensus from non‐standardized models

**Figure 1:**
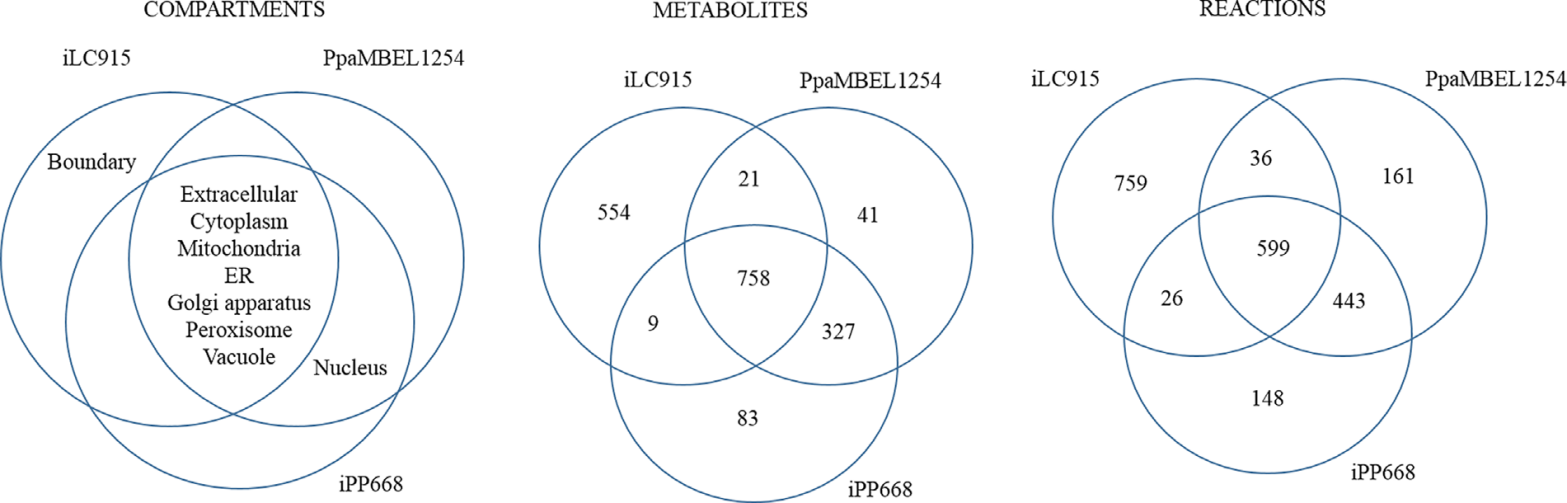
Comparison of the existing GEMs of *K. phaffii*. The numbers in the intersection sets of the models for the metabolites and reactions represent the number of entities commonly found either two or all of the models. The remaining metabolites and reactions were classified as unique to each model

The lack of standard IDs for metabolites, enzymes, and reactions in the three available metabolic models for *K. phaffii* (Caspeta et al., 2012; Chung et al., 2010; Sohn et al., 2010) complicates both their re‐use and the task of constructing a consensus model. In order to obtain a reusable model, we started to reconstruct the metabolic network of *K. phaffii* by comparing the existing independently constructed networks. Each construction had eight compartments, where the cell‐boundary compartment was unique to iLC915, while the nucleus was included only in PpaMBEL1254 and iPP668 (Figure 1). All nine compartments were included in our reconstruction. We found 1,793 metabolites that were compartmentalized into nine different compartments in these three extant models and ca. 42% of them were found in all three models (Figure 1, File S2). There were 2,132 reactions, 28% of which were present in all 3 networks (Figure 1, Table S2). It should be noted that not all of the representations of these reactions were exactly same; the differences involving currency metabolites, directionality, and compartmentation. For instance, of the 597 reactions that were common to all three models, only 276 reactions were exactly same. This fact, alone, illustrates the need for manual intervention in order to assess the similarity between metabolic models when the standard reaction ID's are not provided.

An initial core network was constructed considering only reactions found in at least two of the three models. Even some of these were omitted due to the lack of any evidence for their existence in *K. phaffii* or due to the lumping together of multiple reactions (e.g., in fatty acid metabolism) in the iPP668 and PpaMBEL1254 models, which precluded the assignment of standard reaction IDs. Following this manual cross‐checking exercise, our initial core network comprised 915 reactions and 965 metabolites. Further analysis revealed gaps and a high proportion (∼45 %) of dead‐end metabolites, and so required further manual curation.

### 3.2 Deficiencies in lipid metabolism and compound transport were the main contributors to poor network connectivity

There were three main reasons for the high proportion of dead‐end metabolites in the initial core network: missing pathways, inconsistencies in the compartmentation of the network, and insufficient representation of intracellular transport processes. Fatty acid metabolism, which (as indicated earlier) was not well defined in the initial core network, was the principal contributor to gaps in the network. The level of detail in the representation of the fatty acid biosynthesis, elongation, and degradation pathways varied between the three extant models. This required the de novo reconstruction of these pathways, using the information for *K. phaffii* metabolism in KEGG (Kanehisa et al., 2016; Kanehisa & Goto, 2000). Based on published experimental evidence for the generation of acyl groups up to a C_16_ chain length by the mitochondrial FAS pathway in yeast (Hiltunen et al., 2009), the fatty acid biosynthesis reactions involving acyl carrier protein (ACP) were assigned to the mitochondrial and cytosolic compartments. Fatty acid elongation reactions involved in the elongation of fatty acids up to C_24_ were incorporated into the endoplasmic reticulum in the model, whereas the reactions involved in fatty acid degradation were localized to the peroxisome. Further gap‐filling was conducted using information on gene orthology with *S. cerevisiae*.

The inconsistencies in the assignment of reactions involved in glycerophospholipid metabolism to compartments also resulted in the formation of dead‐end metabolites. The localization assignments were performed using the GO (Ashburner et al., 2000) cellular component terms of the genes encoding the enzymes that catalyze those reactions. Since the biomass representation in the three pre‐existing models did not include sphingolipid content, sphingolipid metabolism was not connected with the rest of the metabolism. Tomàs‐Gamisans, Ferrer, and Albiol (2016) recently published an integrated genome‐scale metabolic model (iMT1026) of *K. phaffii*, where they updated both sphingolipid metabolism and the sphingolipid content of the biomass equation based on the literature information. Their representation of sphingolipid metabolism, as reported in iMT1026, was incorporated into the Kp.1.0 model.

The other major cause of dead‐end metabolites was the absence of transport reactions to enable those metabolites to move between organelles. The localization of all reactions involving dead‐end metabolites was cross‐checked and either the localization assignments were amended or the required transport reactions were included, as necessary.

### 3.3 Implementation of gene‐reaction associations to the executable K. phaffii model to allow automated gene deletion studies

After the gap‐filling step, the final version of the metabolic network contains 1,424 reactions (including the 12 different biomass formation reactions and 2 different reactions corresponding to recombinant protein production) and 1,221 metabolites. Gene‐reaction associations were built on this network. Comparison of the gene‐reaction associations given in the existing models revealed crucial differences in these associations and also in the logical rules applied when multiple genes were associated with a single reaction. There were instances where the reactions in the glycolysis, amino‐acid biosynthesis, purine metabolism, pyrimidine metabolism, and fatty acid elongation pathways were associated with a single enzyme in iPP668 but with more than one enzyme in iLC915 (File S2). For Kp.1.0, we used the reaction‐enzyme associations in KEGG database (Kanehisa et al., 2016; Kanehisa & Goto, 2000) to assign gene‐reaction relationships and formulate rules to determine gene essentiality. Sub‐cellular localization of proteins and gene‐deletion phenotypes were based on orthology with *S. cerevisiae*. By these means, we determined that 1,135 of the 1,424 reactions in Kp.1.0 were associated with 720 *K. phaffii* genes.

### 3.4 Ability of the model to predict gene essentiality

The model was evaluated in terms of its success in predicting whether or not null mutants (deletants) were viable. Since there is, as yet, no comprehensive collection *of K. phaffii* deletants, empirical data on mutant phenotypes are not available for this species. Instead we assigned phenotypes to null mutants of *K. phaffii* genes based on their orthology with *S. cerevisiae* genes. Among the 720 genes involved in the model, 612 genes have an ortholog in *S. cerevisiae* (Sonnhammer & Östlund, 2015). In all, 26% of the 720 genes represented in the model were predicted to be essential (File S3). This percentage was 29% for the genes having *S. cerevisiae* orthologs. Based on the deletion mutant phenotypic data reported for orthologs of these genes in *S. cerevisiae* (Nash et al., 2007), the Kp.1.0 model could predict 80% of the viable and 57% of the inviable phenotypes (Table 3). The stoichiometric model of the *S. cerevisiae* metabolic network (Yeast 7) (Aung, Henry, & Walker, 2013) was also used to predict viable/lethal phenotypes of *S. cerevisiae* strains bearing deletions of the genes having *K. phaffii* orthologs. Using the *S. cerevisiae* Y7.6 model, the viable and lethal phenotypes could be predicted correctly in 82% and 70% of the cases, respectively (Table 3, File S3). A similar analysis could only be conducted with iLC915, since it is the only existing model that contains gene‐reaction associations in the SBML version. In this case, the viable phenotypes were correctly predicted in 90% of the cases whereas only 11% of lethal phenotypes could be correctly predicted (Table 3).

**Table 3:** A comparison of the predictive ability of Kp.1.0 and Y7.6

	Kp1.0	Y.7.6	iLC915
# of genes	720	909	915
Essential genes	26%	17%	9%
# of orthologous genes in GEM	612	517	726
Essential genes (among orthologs)	29%	21%	10%
# of TP	370	338	483
# of FP	62	32	171
# of TN	82	74	21
# of FN	95	73	51
Sensitivity	80%	82%	90%
Specificity	57%	70%	11%
Positive predictive value	86%	91%	74%
Negative predictive value	46%	50%	29%
% Correct prediction	74%	80%	69%

Next, we checked whether or not the orthologs of the 608 genes in Kp.1.0 have paralogs within the *S. cerevisiae* genome. Of the 608 genes in Kp.1.0 with a *S. cerevisiae* ortholog, 76 genes were identified to have a second, paralogous, copy that arose from the whole‐genome duplication (File S4). For 16 of those genes, model simulations using Kp.1.0 predicted an inviable phenotype, whereas their yeast orthologs were reported to be viable. This is indicative of the lower level of genetic redundancy in the *K. phaffii* genome, and is entirely congruent with the fact 11 of those 16 genes were reported to have a synthetic lethal interaction with their paralogs in *S. cerevisiae* (Stark et al., 2006).

### 3.5 Ability of the model to predict K. phaffii physiology

After benchmarking our model by predicting gene essentiality, we next investigated the ability of the Kp.1.0 model to predict the physiological performance of *K. phaffii*. In this case, in contrast to predicting gene essentiality, we were able to use published experimental data on the growth characteristics, at different oxygen levels (Carnicer et al., 2009), of both wild‐type *K. phaffii* cells and those producing a recombinant protein. In addition, data were available comparing the growth of wild‐type cells on either glucose or methanol/glycerol (Rußmayer et al., 2015). These empirical data were used to constrain the model as shown in Table 1, and the growth rate and CO_2_ exchange rate (CER) were predicted employing condition‐specific biomass compositions, in the case of Carnicer et al., and using the biomass composition of wild‐type cells under normoxic conditions for the other dataset. The error rates were within a 1–15 % range with one exception, where the CO_2_ exchange rate (CER) for wild‐type, glucose grown‐cells was predicted to be ∼25% higher than the reported experimental value (Table 4).

**Table 4:** Model predictions on growth characteristics

	Experimental values		Predicted values	
	Growth rate (hr^−1^)	CER (mmol/gCDW/hr)	Growth rate (hr^−1^)	CER (mmol/gCDW/hr)
Wild type				
Normoxic	0.1	2.43	0.10	2.62
Oxygen‐limited	0.1	2.55	0.09	2.71
Hypoxic	0.1	3.21	0.09	3.37
Fab producing				
Normoxic	0.1	2.52	0.09	2.70
Oxygen‐limited	0.1	2.68	0.09	2.88
Hypoxic	0.1	2.94	0.09	3.16
Wild type^a^				
Grown on Glu	1	2.11	0.10	2.66
Grown on Met + Gly	0.1	1.86	0.10	2.15

^a^ The model predictions provided in this table were obtained when the biomass composition for cells grown under normoxic conditions for wild‐type cell were used.

### 3.6 The impact of biomass composition on predictions made with Kp.1.0

Using a methanol and glycerol mixture as a co‐carbon source is widely employed cultivation strategy for *K. phaffii*, but there are no experimental data on the biomass composition of wild‐type cells grown under this condition. For this reason, we decided to use this culture strategy as a test case with which to evaluate the effect of biomass composition on model predictions and flux distributions. We performed in silico experiments in which wild‐type cells were grown on methanol/glycerol (8.5/49 g/g) in carbon‐limited chemostat culture at a dilution rate of 0.1 hr^−1^. Glycerol, methanol and oxygen uptake rates were constrained according to the data of (Rußmayer et al., 2015) and the simulations were repeated using 12 different representations of biomass composition. Both growth rate and CO_2_ exchange rate predictions were used to evaluate the predictive accuracy of the Kp.1.0 model. The % errors in predicting the CO_2_ exchange and growth rates were, in all cases, below 25% and 15%, respectively (Table 5). To test the effect of biomass representation on the predicting the phenotypes of null mutants, simulations for single‐gene deletants were conducted using the different biomass composition representations. In no case did the biomass composition have any impact on the mutant phenotype.

**Table 5:** Biomass effect on model prediction

Biomass content data under description of condition	Predicted CER (mmol/gCDW × hr) (experimentally determined: 1.86 mmol/gCDW × hr)	Predicted growth rate (hr^−1^) (experimentally determined: 0.1 hr^−1^)
1. Wild type—normoxic	2.15	0.10
2. Wild type—oxygen limited	2.18	0.09
3. Wild type—hypoxic	2.21	0.09
4. Fab producing—normoxic	2.14	0.09
5. Fab producing—oxygen limited	2.24	0.09
6. Fab producing—hypoxic	2.28	0.10
7. 80/20 glycerol/methanol—*D* = 0.05 hr^−1^	2.23	0.10
8. 60/40 glycerol/methanol—*D* = 0.05 hr^−1^	2.16	0.11
9. 40/60 glycerol/methanol—*D* = 0.05 hr^−1^	2.17	0.10
10. 80/20 glycerol/methanol—*D* = 0.16 hr^−1^	2.03	0.11
11. 60/40 glycerol/methanol—*D* = 0.16 hr^−1^	2.02	0.11
12. 40/60 glycerol/methanol—*D* = 0.16 hr^−1^	2.16	0.11

**Figure 2:**
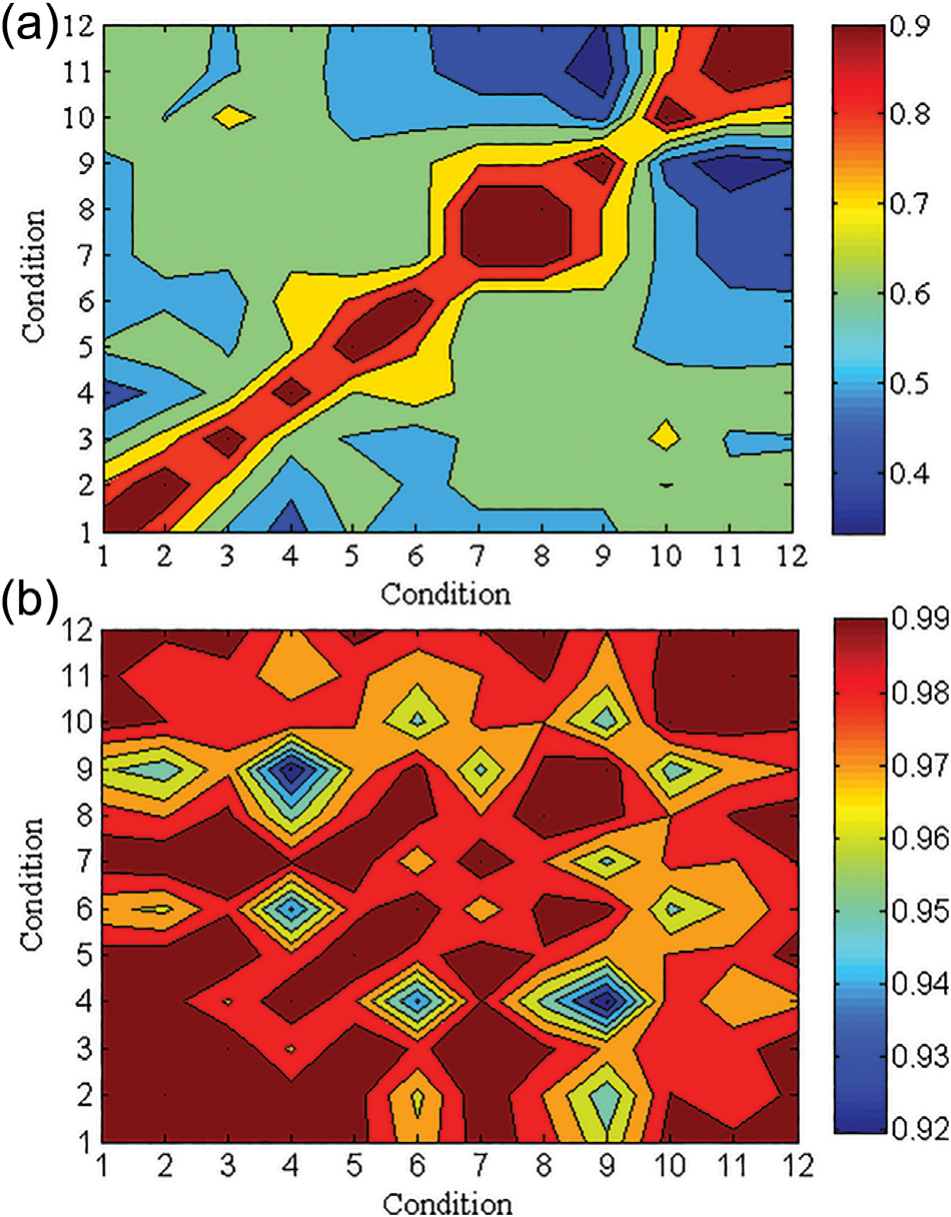
Correlations between flux distributions and corresponding biomass compositions. The plot represents (a) the correlations between the flux distributions when the simulation were conducted using different biomass compositions (b) the correlation between the corresponding biomass compositions. The labels in the abscissa and ordinate corresponds to conditions where, wild type cells grown under normoxic (1), oxygen limited (2), hypoxic environment (3), Fab producing cells grown under normoxic (4), oxygen limited (5) and hypoxic environment (6), cells grown at a dilution are of 0.05 hr^−1^ using 80/20 glycerol/methanol (7), 60/40 glycerol/methanol (8), 40/60 glycerol/methanol (9) as the carbon source and cells grown at a dilution are of 0.16 hr^−1^ using 80/20 glycerol/methanol (10), 60/40 glycerol/methanol (11), 40/60 glycerol/methanol (12) as the carbon source. The color bar indicates values of the Pearson correlation coefficient: an increase from red to blue means transition from high correlation to low correlation

Given this result, we decided to investigate the effect of biomass composition on the flux distributions. To achieve this, we constructed two correlation matrices showing: (1) correlations between the flux distributions obtained when the biomass compositions measured experimentally under different conditions were used (Figure 2a), and (2) correlations between the coefficients of the macromolecules of biomass content under these conditions (Figure 2b). We found that, even when two different growth conditions produced very similar (experimentally determined) biomass compositions, the cells grown under those two conditions could have widely different in silico flux distributions.

Flux variability analysis was conducted to identify the reactions that display the greatest variability, and the impact of the biomass composition on these changes was investigated. It was observed that the highly variable reactions were catalyzed by enzymes involved in phosphorus, carbohydrate, and ribonucleoside diphosphate metabolism; these associations were significant (*p*‐value < 0.01). We found that the reactions that show high variability did not change when different biomass representations were used.

**Figure 3:**
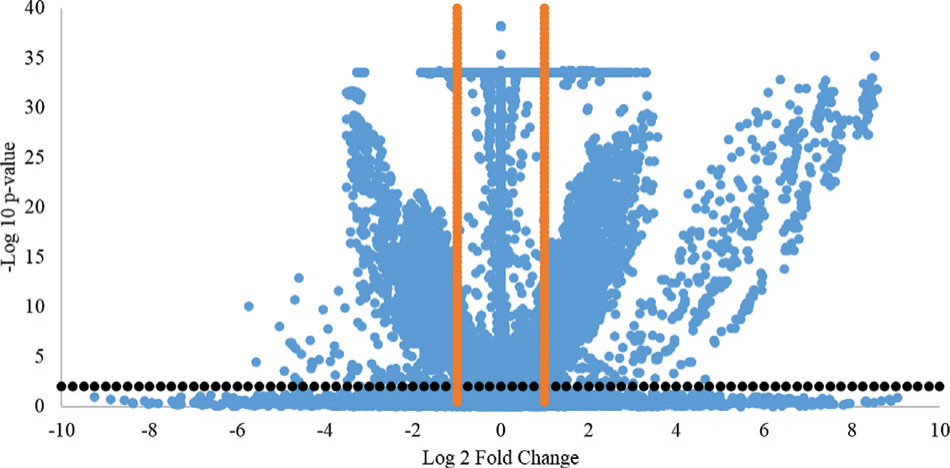
Volcano plot of the flux distributions obtained using different biomass composition reactions. The plot represents the comparison of the flux values for every possible combinations of 12 different distributions. The *x*‐axis shows the log 2 fold changes and the *y*‐axis shows the log 10 of the *p*‐values. Each blue dot corresponds to a reaction for a specific comparison. The orange dots represents the threshold for fold change values (FC > 2) and the black dots represent the threshold used for *p*‐values (*p*‐value < 0.01)

The significance of the change in flux values was evaluated, for each reaction, by comparing each condition with every other. For a sample size of 100, we found that 433 out of the 1,474 reactions in Kp.1.0 showed a significant change in at least one of the comparisons (*p*‐value < 0.01, FC > 2) (Figure 3, File S5). There was no difference in the number of significantly changed fluxes between the conditions that have significantly different biomass compositions (*p*‐value < 0.1) and those that showed no significant difference in biomass content.

Both the correlation and significance analyses indicated that the effect of biomass representation on flux distribution could not be predicted. There were 215 genes associated with the reactions whose flux was significantly changed by altering biomass composition, independent of the sample size. GO analysis showed that this gene set was significantly enriched for the following biological process terms: steroid biosynthesis, lipid biosynthetic, cellular alcohol metabolic process, and oxidation–reduction (*p*‐value < 0.01). When the same analysis was conducted with a sample size of 100 using iLC915, incorporating the same biomass equations, it was observed that 693 reactions, which were associated with 452 genes were significantly changed. These genes were identified to be enriched only with the process terms cellular lipid metabolic process and cell cycle, and cellular catabolism (*p*‐value < 0.05) (File S6). The same analysis was repeated for sets of flux distributions of various sample sizes of 50, 1,000, and 10,000, in order to identify any possible biases for sample size, and the results of the analysis indicated that the number of reactions whose fluxes were significantly different and the number of genes associated with those reactions, remained within ±1.25% and ±2.50% of one another, respectively; thus no size‐dependent trends were observed. More than 92% of both the reactions and the genes associated with these reactions were common in all sets and consequently the GO Process Term enrichment analysis highlighted the same processes for all of them, demonstrating the robustness of this analysis (Table S1, File S5).

Observation of the impact of biomass representation on flux distributions prompted us to investigate the effect of the biomass content on target identification. Overexpression targets to improve production of human copper/zinc superoxide dismutase (hSOD) were predicted using 12 different biomass representations and, in all, 59 genes were identified as candidates. Among these 59 genes, 37 were classified as targets in all cases, whereas 22 were identified only using some of the biomass representations. The genes encoding the enzymes catalyzing those reactions identified to have an increased flux in all analyses were enriched with histidine and lysine metabolic processes; whereas the genes catalyzing the reactions that showed condition‐dependent increase were enriched with glutamine family amino‐acid biosynthetic process (*p*‐value < 0.01).

The effect of the biomass composition on predicting knockout targets to improve hSOD production was evaluated using RobOKoD (Stanford et al., 2015), which allows the comparison of the score‐based rankings of the potential knock‐out targets. Thirteen genes were identified as potential knockout targets (File S7). Eight of these genes were identified in all cases, whereas five of them were biomass dependent. These genes were not significantly enriched with any process term. Comparison of the rankings of the candidates revealed that there were some cases, where a gene that was identified as the best candidate for some cases was ranked down the list for another case. For instance, PAS_chr1‐4_0149 was identified as the best candidate when the simulations were conducted using either the biomass representation for normoxic conditions or when cells were grown at a dilution rate of 0.16 hr^−1^, but this gene was ranked lower when the results were obtained using the biomass representation for wild type cells grown under hypoxic conditions (File S7).

## 4 DISCUSSION

If GEMs are to fulfill their potential as tools to enable strain design in synthetic biology, they must satisfy at least four essential criteria. First, a GEM must be executable in order to allow analyses and simulations to be performed. Second, the model must be readily manipulable by the user, both to enable analyses to be performed and allow the entire research community to participate in model curation to ensure the GEM is as comprehensive, accurate, and up‐to‐date as possible. This requirement means that the metabolic model must be presented in a standard format, of which the most widely used is the Systems Biology Mark‐up Language (Hucka et al., 2003). Fulfillment of the second criterion also demands all metabolites, enzymes, and genes are unambiguously annotated with database IDs. The use of standard formats and IDs facilitates the extension of a model, as well as comparisons of different versions of the same model or different GEMs for the same species (Ravikrishnan & Raman, 2015). Thirdly, if a GEM is to be used to identify genes to target in strain design, it must incorporate unambiguous gene–enzyme assignments. This last requirement is non‐trivial since two or more genes may specify a single enzyme, or a single gene may specify a protein product with more than one enzyme activity. This problem brings us to the final requirement: the GEM must specify the sub‐cellular location in which each reaction occurs or in which each enzyme functions.

We constructed Kp.1.0 as a GEM for the industrial yeast *K. phaffii* since none of the existing models satisfied all these requirements. Our consensus model is provided in SBML format including the relevant systematic IDs for the metabolites and reactions as previously proposed (Herrgård et al., 2008; Ravikrishnan & Raman, 2015) in order to permit reusability and improvement by the community. The currently available GEMs for *K. phaffii* were first reconciled and then extensive manual curation was carried out to generate Kp.1.0. The manually curated gene‐reaction associations in the SBML version of the model will allow synthetic biologists to carry out target identification. A recent reconstruction of a *K. phaffii* GEM (Tomàs‐Gamisans et al., 2016) did not consistently incorporate the AND/OR relationships into the executable version of the model when a reaction is catalyzed more than one gene product.

Kp.1.0 was validated through evaluating its success in predicting whether single‐gene null mutants would be viable or inviable. The essentiality information on *S. cerevisiae* used in this validation exercise exploits the orthology between *K. phaffii* and *S. cerevisiae* genes, since a deletion collection of *K. phaffii* is not yet available. Conservation of gene essentiality between yeast species is well known and is found even between the extremely distantly related yeast species, *Schizosaccharomyces pombe* and *S. cerevisiae* (Kim et al., 2010). Kp.1.0 was found to have a comparable success to that of *the S. cerevisiae* GEM Yeast v.7 (Aung et al., 2013) in predicting the viability of the null mutants. Moreover, some predictions, which were considered as false‐negatives based on orthology information, could be explained by the fact that, unlike *S. cerevisiae*, *K. phaffii* has not undergone an ancestral whole‐genome duplication (Förster, Halbfeld, Zimmermann, & Blank, 2014). Because of this, genes that are duplicated in *S. cerevisiae* are present in only a single copy (and are, therefore, essential) in *K. phaffii*.

In order to confidently and unambiguously define gene‐reaction associations, putative genes and genes with unknown function, which comprise 5–10% of the genes included in the other *K. phaffii* GEMS were omitted from Kp.1.0. Furthermore, during the construction of Kp.1.0, enzymes catalyzing reactions that led to an increase in the number of dead‐end metabolites (e.g., in the glycosylphosphatodylinositiol (GPI) anchor biosynthesis pathway, and in lipoic acid metabolism) were omitted, together with their cognate genes. This was done because as the number of genes associated with dead‐end reactions increases, the number of genes predicted to be non‐essential will also increase, irrespective of the model structure. The low specificity of the iLC915 GEM in predicting gene essentiality is thought to be a consequence of this fact (Table 3).

The biomass composition of microbial cells is bound to vary between different physiological conditions (e.g., different carbon sources, nitrogen limitation, pH, aeration, temperature, mode of cultivation, genetic manipulation; Aguilar‐Uscanga & Francois, 2003; Carnicer et al., 2009; Jordà et al., 2014) such that we have previously referred to biomass composition as the “elephant in the room” of metabolic modeling (Dikicioglu, Kırdar, & Oliver, 2015). A great advantage to modeling the metabolic network of *K. phaffii* is that, in contrast to *S. cerevisiae*, extensive data on biomass composition of this yeast have been collected under different experimental and genetic conditions. This provided a great opportunity to study the effect of biomass composition on the success of the Kp.1.0 model in predicting growth characteristics, flux distributions, gene essentiality, and the identification of the targets to be engineered for production of a specific r‐protein.

These analyses showed that growth characteristics could be predicted with an error rate changing between 2% and 22% depending on the biomass composition used in Kp.1.0. It was observed that the varying the representation of biomass composition did not affect the prediction of gene essentiality. On the other hand, when the flux distributions were investigated, it was seen that the wiring of some parts of the metabolic network changed greatly depending on the biomass composition. The analyses of these significantly altered fluxes, and the genes encoding the enzymes that catalyze the reactions involved, revealed the impact of biomass composition was not random; rather, it affected specific domains of metabolism, including lipid and secondary alcohol biosynthesis and oxidation‐reduction processes. A similar analysis conducted with iLC915, the only other *K. phaffii* GEM for which gene‐reaction associations are available, also highlighted the lipid metabolic processes as being sensitive to changes in biomass composition. This suggests that the specific effects of changes in biomass composition are not peculiar to a particular network reconstruction but, rather, are intrinsic to the metabolism of this industrial yeast species.

The representation of biomass content also affected the predictions on potential targets to be engineered for improving r‐protein production when flux distribution based approaches such as FSEOF approach were used. Identification of the overexpression targets to improve hSOD production using Kp.1.0 model employing 12 different biomass representations showed that 37% of the targets were classified as targets conditionally. Moreover, further investigation of the effect of biomass representation on predicted beneficial genetic interventions revealed that not only different reactions might be identified as candidates but also the rankings of the candidate targets altered when the biomass content was changed in the model. These results showed that biomass content was a key player in target identification using metabolic modeling.

We believe that the Kp.1.0 genome‐scale metabolic model represents a useful tool with which to design more productive strains of *K. phaffii* It is suitable to conduct such analyses and has been made, available to the community through the BIOMODELS database (Chelliah et al., 2015) and assigned the identifier MODEL1703150000. Since obtaining a consensus model is only possible with community effort, we believe that Kp.1.0 can form a basis for future improvements of both the basic model and its modification for specific applications in biotechnology (Irani, Kerkhoven, Shojaosadati, & Nielsen, 2016).

## Supporting information

Additional Supporting Information may be found online in the supporting information tab for this article.

**Table S1**. Effect of Sample Size on Significance Analysis

Supporting Information.S2

Supporting Information.S3

Supporting Information.S4

Supporting Information.S5

Supporting Information.S6

Supporting Information.S7
