# Supplementary material for: Metabolic modeling to identify engineering targets for Komagataella phaffii: The effect of biomass composition on gene target identification
Source: Biotechnol Bioeng. 2017 Aug 15;114(11):2605–15. doi: 10.1002/bit.26380 (PMC5659126; doi:10.1002/bit.26380)
Supplement: Supplementary file 1 — Table S1. Effect of Sample Size on Significance Analysis [file BIT-114-2605-s001.docx]

Table S1. Effect of Sample Size on Significance Analysis

| **# of Randomization** | **# of Significant Reactions** | **# of Genes associated with significant reactions** |
| --- | --- | --- |
| 50 | 431 | 235 |
| 100 | 433 | 227 |
| 1000 | 439 | 236 |
| 10000 | 429 | 229 |
